# The relationship between the BRAF^V600E^ mutation in papillary thyroid microcarcinoma and clinicopathologic factors

**DOI:** 10.1186/1477-7819-11-291

**Published:** 2013-11-14

**Authors:** Sun Yi Choi, HeonSoo Park, Myung Koo Kang, Dong Kun Lee, Kang Dae Lee, Hyoung Shin Lee, Sung Won Kim, Eun Nam Lee, Jong Chul Hong

**Affiliations:** 1 Dong-A University College of Medicine, 3-1 Dongdaeshin-Dong, Busan, Seo-Gu 602-715, South Korea; 2 Department of Otolaryngology-Head and Neck Surgery, Dong-A University of College of Medicine, 3-1 Dongdaeshin-Dong, Busan, Seo-Gu 602-715, South Korea; 3 Department of Otolaryngology-Head and Neck Surgery, Kosin University College of Medicine, Am-Nam Dong 34, Busan, Seo-Gu 602-702, South Korea; 4 Department of Nursing, Dong-A University College of Medicine, Busan, South Korea

**Keywords:** BRAF mutation, Papillary thyroid microcarcinoma, Prognosis

## Abstract

**Background:**

The BRAF^V600E^ mutation, which accounts for about 60–80% papillary thyroid carcinoma(PTC), has been identifiedas a prognostic marker for risk stratification of PTC patients. However, the BRAF^V600E^ mutation as a prognostic marker in papillary thyroid microcarcinoma (PTMC) is unclear.

**Methods:**

We performed a retrospective review of 101 patients who underwent surgery for PTMC. We studied the prevalence of the BRAF^V600E^ mutation. The associations between the BRAF^V600E^ mutation and clinicopathologic characteristics were analyzed.

**Results:**

The BRAF^V600E^ mutation was observed in 72 patients (71.3%). There was no statistically significant correlation in age, gender, multifocality, extrathyroidal extension, presence of Hashimoto thyroiditis, and lymph node metastasis between the BRAF^V600E^ mutant group and wild group.

**Conclusions:**

The BRAF^V600E^ mutation is not significantly associated with prognostic factors in PTMC.

## Background

Thyroid carcinoma is the most common endocrine malignancy and its prevalence is increasing. Recent extensive use of thyroid ultrasound has led to the increased detection of non-palpable thyroid nodules. Papillary thyroid carcinoma (PTC) is the most common type of endocrine malignancy followed by follicular thyroid carcinoma. The prevalence of PTC is rapidly increasing and currently accounts for >95% of all thyroid carcinomas in Korea [1]. Papillary thyroid microcarcinoma (PTMC), a form of PTC, is defined by the World Health Organization as a tumor measuring 1 cm or less in its greatest dimension. Based on the high incidence of PTMC in autopsy studies, its prognosis is generally favorable and sometimes it is truly indolent and non-progressive [2]. However, it has been reported that PTMC may have a metastatic potential similar to that of their clinically detectable counterparts [3-5]. Thus, estimate of prognosis as well as detection of PTMC has become an important issue.

The BRAF^V600E^ mutation, which accounts for about 60–80% of PTCs, has been identifiedas a promising prognostic marker for risk stratification of PTC patients in Korea [4]. However, uncertainties still exist due to the discordance of studies. Some studies show a significant relationship between the BRAF^V600E^ mutation and the high-risk clinicopathologic characteristics of PTC [4,6]. However, other studies have failed to find a significant association between the BRAF^V600E^ mutation and high-risk clinicopathologic characteristics [5,7]. With regards to PTMC, studies show no significant correlation [7]. Nevertheless, considering its nature, further study of the characteristics of PTMC through BRAF^V600E^ mutational status analysis is needed. We investigated the prevalence of the BRAF^V600E^ mutation andanalyzed the relationship between the BRAF^V600E^ mutation and clinicopathologic factors in PTMC.

## Methods

### Patients

The study included review of 253 patients who had undergone thyroid cancer surgery between June 2011 and July 2012 at the department of Otolaryngology-Head and Neck surgery, Dong-A Medical Center, Busan, Korea. Most patients underwent total thyroidectomy with general anesthesia. In case of unifocal intrathyroidal microcarcinoma with no evidence of cervical lymph node metastasis in preoperative ultrasonography, we conducted thyroid lobectomy. Central compartment neck dissection was performed when an enlarged lymph node or invasion of the thyroid capsule was detected during surgery. Modified radical neck dissection was conducted in cases of lateral lymph node metastasis. Of these 253 patients,101 patients diagnosed withconventional PTMC were enrolled in the study. Informed consent for the evaluation of BRAF^V600E^ mutation was obtained from all PTMC patients. We studied the prevalence of the BRAF^V600E^ mutation and evaluated its correlations with diverse clinicopathologic features of PTMC.

### DNA extraction and the BRAF^V600E^ mutation analysis

We performed the BRAF^V600E^ mutation analysis on paraffin embedded sections of primary tumors obtained after thyroidectomy. Genomic DNA was extracted from 10 μm-thick sections of 10% neutral formalin-fixed paraffin-embedded tumor tissue blocks using the High Pure PCR Template Preparation Kit (Roche Applied Science, Germany). The concentration and purity of the extracted DNA were determined by a NanoDrop ND-1000 spectrophotometer (NanoDrop Technologies, USA). The extracted DNA was stocked at −20°C until further use.

**Figure 1:**
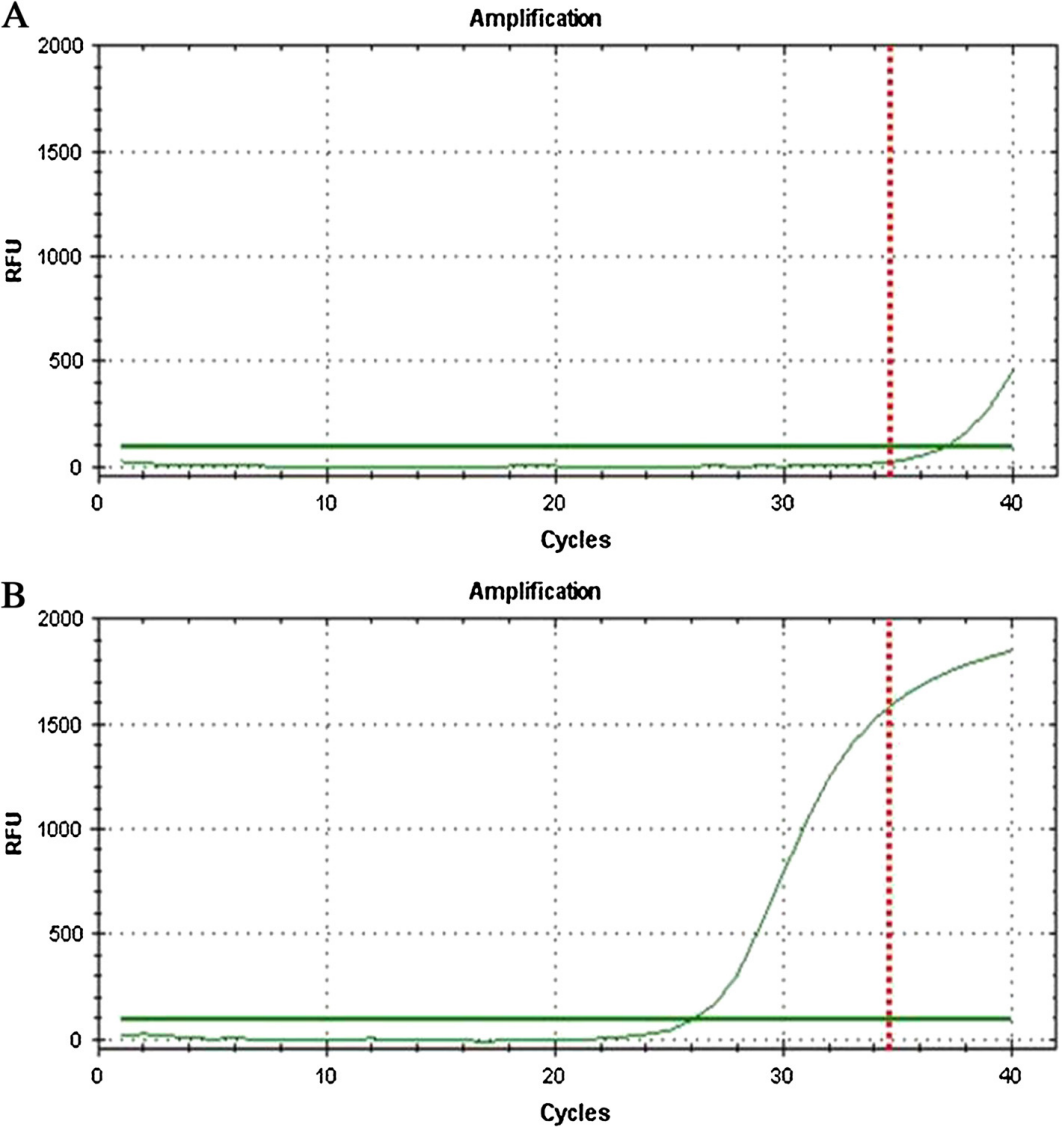
**The assays for the detection of the BRAF**^**V600E**^**mutation. (A)** In a patient with left thyroid nodule, the ΔCt-1 was measured as-2.17, and was identified as wild-type. **(B)** In another patient with right thyroid nodule, ΔCt-1 was measured as 8.86, and was identified as mutant type.

The assays for the detection of the BRAF^V600E^ mutation wereperformed usingthe PNAClamp^TM^BRAF Mutation Detection kit (Panagene, Inc.,Daejeon, Korea). All reactions were done in 20-μLvolumes using template DNA, primer and peptide nucleic acid(PNA) probe set, and PCR master mix. All needed reagents were included with the kit. Real-time PCR reaction of PNA-mediated clamping PCR was performed using a CFX 96 (Bio-Rad, USA). PCR cycling conditions were a 5 min hold at 94°C followed by 40 cycles of 94°C for 30 sec, 70°C for 20 sec, 63°C for 30 sec, and 72°C for 30 sec. In this assay, PNA probes and DNA primers are used together in the clamping reaction. Positive signals are detected by intercalation of fluorescent dye. The PNA probe sequence is complementary to wild-type DNA to suppress amplification of wild-type target, thereby enhancing preferential amplification of mutant sequences by competitively inhibiting DNA primer binding to wild-type DNA. PCR efficiency was determined by measuring the threshold cycle (Ct) value. Ct values for the control and mutation assays were obtained by observing the amplification plots. The delta Ct (ΔCt) value was calculated as follows, ensuring that the sample and Standard Ct values werefrom the tested sample and clamping control sample: [Standard Ct]-[Sample Ct] = ΔCt. The cut-off ΔCt was defined as 2.0 for the BRAF^V600E^ mutation (Figure 1).

### Statistical analysis

To evaluate the association between BRAF^V600E^ mutation and prognostic variables, we used χ^2^or Fisher’s exact test and logistic regression using the SPSS 18.0 program (SPSS Inc., Chicago, IL, USA). Statistical significance was defined as *P* < 0.05.

## Results

All included patients had PTMC confirmed on the surgical specimen. Of 101 patients, BRAF^V600E^ mutation in PTMC was observed in 72 patients, indicating a prevalence of 71.3%. The number of patients aged ≥45 years were 76[mutant: 56 (77.8%), wild: 20(69.0%)] and showed no statistical significance. The female sex was dominant in PTMC patients (89.1%); however, there was no statistical significance with BRAF^V600E^ mutation. There were 31 patients with multifocality [mutant: 22(30.6%), wild: 9(31.0%)], 53 patients with extrathyroidal extension [mutant: 37(51.4%), wild: 16(55.2%)], and 22 patients with combined Hashimoto’s thyroiditis [mutant: 14(19.4%), wild: 8(27.6%)]. These three factors were not significantly related. There were 48 T1a patients [mutant: 36(50.0%), wild: 12(41.4%)], 52 T3 patients [mutant: 35(48.6%), wild: 17(58.6%)], and 1 T4a patient [mutant: 1(1.4%), wild: 0(0%)]. There were 18 patients with nodal metastasis; among these, 11 were N1a [mutant: 8 (11.1%), wild: 3 (10.3%)] and 7 were N1b [mutant: 3 (4.2%), wild: 4 (13.8%)]. The T and N staging were not significantly related to BRAF^v600E^mutation (Table 1). Distant metastasis to the lung was found in one patient with wild type and it was not significantly relevant with BRAF^v600E^.

**Table 1:** **Relationship between the BRAF**^**V600E**^**mutation and clinicopathologic factors in papillary thyroid microcarcinoma**

	**Total, n(%)**	**BRAF**^**V600E**^**mutation, n (%)**			
		**Mutant (n = 72)**	**Wild (n = 29)**	***χ***^**2**^	***P*** **value**
Age				0.862	0.353
<45 years	25(24.8)	16(22.2)	9(31.0)		
≥45 years	76(75.2)	56(77.8)	20(69.0)		
Gender				0.669	0.413
Female	90(89.1)	63(87.5)	27(93.1)		
Male	11(10.9)	9(12.5)	2(6.9)		
Multifocality				0.002	0.962
No	70(69.3)	50(69.4)	20(69.0)		
Yes	31(30.7)	22(30.6)	9(31.0)		
Extrathyroidal extension				0.119	0.73
No	48(47.5)	35(48.6)	13(44.8)		
Yes	53(52.5)	37(51.4)	16(55.2)		
Hashimoto thyroiditis				0.804	0.37
Not combined	79(78.2)	58(80.6)	21(72.4)		
Combined	22(21.8)	14(19.4)	8(27.6)		
T staging				1.128	0.569
T1a	48(47.5)	36(50.0)	12(41.4)		
T3	52(51.5)	35(48.6)	17(58.6)		
T4a	1(1.0)	1(1.4)	0(0)		
Nodal metastasis				2.973	0.226
N0	83(82.2)	61(84.7)	22(75.9)		
N1a	11(10.9)	8(11.1)	3(10.3)		
N1b	7(6.9)	3(4.2)	4(13.8)		
Distant metastasis				2.508	0.113
No	100(99.0)	72(100.0)	28(96.6)		
Yes	1(1.0)	0(0.0)	1(3.4)		

In summary, as shown in Tables 1 and 2, there were no statistically significant correlationswith regards to age, gender, multifocality, extrathyroidal extension, presence of Hashimoto thyroiditis, and lymph node metastasis between the BRAF^V600E^ mutant and wild-type groups.

**Table 2:** **Summary of logistic regression for variables predicting the BRAF**^**V600E**^**mutation in PTMC**

**Variables**	**B**	**SE**	**Wald**	**Df**	***P *** **value**	**Odds ratio**	**95% confidence interval**	
							**Low**	**High**
Age (1 = <45, 0 = ≥45^*^)	−0.541	0.535	1.024	1	0.312	0.582	0.204	1.661
Gender (1 = female, 0 = male^*^)	−0.673	0.854	0.621	1	0.431	0.510	0.096	2.720
Multifocality (1 = no, 0 = yes^*^)	−0.007	0.512	0.000	1	0.989	0.993	0.364	2.707
Extrathyroidal extension (1 = no, 0 = yes^*^)	0.101	0.485	0.044	1	0.835	1.106	0.428	2.860
Hashimoto thyroiditis (1 = not combined, 0 = combined^*^)	0.481	0.532	0.817	1	0.366	1.617	0.570	4.586
Nodal metastasis (Ib)	-	-	2.636	2	0.268	-	-	-
Nodal metastasis (1 = N0, 0 = NIb^*^)	1.384	.855	2.622	1	0.105	3.992	0.747	21.325
Nodal metastasis (1 = NIa, 0 = NIb^*^)	1.303	1.053	1.530	1	0.216	3.680	0.467	29.005
Constant	−0.015	1.230	0.000	1	0.991	0.986	-	-

^*^Reference group.

## Discussion

PTMC belongs to the low-risk well-differentiated PTC group of carcinomas, which are probably of little clinical significance and do not affect patient survival. However, PTMC may be associated with lymph node metastases at presentation and/or loco-regional recurrence during follow-up [8]. Xing et al. investigated the relationship between BRAF^V600E^ mutation and PTC-related mortality in 1,849 patients. The overall mortality was 5.3% vs. 1.1% (mutant vs. wild, *P* < 0.001). They showed the BRAF^V600E^ mutation was significantly associated with increased cancer-related mortality among patients with PTC [9]. In their study, it is uncertain that the BRAF^V600E^ mutation is correlated with poor prognostic factors in PTMC. In the currentstudy, we investigated the incidence of BRAF^V600E^ mutation and the clinicopathologic relationship between mutant and wild-type groupsofPTMC patients.

BRAF mutations are the most common genetic alterations in PTC. Thesemutations activate the RAS/RAF/mitogen-activated protein kinase pathway and result in the malignant transformation of cells. The T1779A point mutation in BRAF exon 15, resulting in a V600E amino acid substitution, is the most common and represents more than 90% of all the mutations found in the BRAF gene [10]. In Korea, the BRAF^V600E^ mutation has been reported to be present in about 60–80% of PTC, which is higher than what is reported in other countries [4-7,10]. In this study, the BRAF^V600E^ mutation was observed in 72 PTMC patients (71.3%). Guan et al. reported that the BRAF^V600E^ mutation was found in 69% of PTC cases in high iodine intake areas and 53% in normal iodine intake areas (*P* < 0.0001) [11]. The authors think that Koreans eat iodine-rich diet, which increases the prevalence of the BRAF^V600E^ mutation in PTC. Further study is needed to evaluate the association between BRAF^V600E^ mutation and the iodine-rich diet in the Korean population.

In our study, we did not find a significant relationship between BRAF^V600E^ mutation and clinicopathologic characteristics such as older age, gender, Hashimoto thyroiditis, T staging, nodal metastasis, multifocality, extrathyroidal extension, and distant metastasis. Based on these results, it seems that the BRAF^V600E^ mutation is not related to the prognostic factors for PTMC thatdetermine the invasiveness of the tumors.

Several studies have shown an association between the BRAF^V600E^ mutation and prognostic factors in PTC. Kebebewet al. reported the BRAF^V600E^ mutation was associated with older age (*P* = 0.0381) [6]. However, Kim et al.[4] and Lee et al. [12] revealed that age was not associated with the BRAF^V600E^ mutation. In our study, the prevalence of mutation in patients aged ≥ 45 years was higher than in wild-type patients (77.8% vs. 69%, *P* = 0.353). However, there was no significant association in patients with old age. Kim et al. [4] and Xu et al. [13] showed the BRAF^V600E^ mutation was associated with male gender. We found a higherprevalence of mutation in the male gender (12.5% vs. 6.9%, *P* = 0.413), althoughthis is not statistically significant due to the small number of male patients. Further, other reports showed no association between the BRAF^V600E^ mutation and male gender [5-7,12]. Lim et al. reported that the BRAF^V600E^ mutation was associated with extrathyroidal extension and multifocality [14]. Kim et al. showed no significant relationship between the BRAF^V600E^ mutation and extrathyroidal extension and multifocality [7]. Our results also showed no relationships (*P* >0.05). However, in the study by Park et al.*,* multifocality was associated with the BRAF^V600E^ mutation [12]. Lim et al. reported a significant association between the BRAF^V600E^ mutation and Hashimoto thyroiditis [14]. In this study, we could not find these significant associations. Several studies have reported that lymph node metastasis was significantly higher in patients withthe BRAF^V600E^ mutation [4,6,12,14]. In our result, lymph node metastasis had no significant association with the BRAF^V600E^ mutation (15.3% vs. 24.1%, *P* = 0.226). Ahn et al. [5] and Kim et al. [7] also found that the lymph node metastasis did not differ significantly between patients with and without the BRAF^V600E^ mutation.

Against expectations, nodal metastasis and distant metastasis showed a slight negative association with BRAF^v600E^ mutation. This result seems to be due to the small sample size andshort follow-up period time. Therefore, oncologic outcomes such as recurrence, metastasis, and survival rate could not be observed. Further, the higher incidence of BRAF^V600E^ mutation in Korea may have had an effect on the study results. Future studies with larger patient groups are needed to evaluate the oncologic outcomes.

## Conclusions

In this study, we have shown that the prevalence of the BRAF^V600E^ mutation was 71.3% in 101 PTMC patients in Korea. The BRAF^V600E^ mutation was not associated with prognostic factors in patients with PTMC.

### Consent

Our study is retrospective study, and all of our patients agreed to us using their medical information.

## Abbreviations

Ct: Threshold cycle; PNA: Peptide nucleic acid; PTC: Papillary thyroid carcinoma; PTMC: Papillary thyroid microcarcinoma.

## Competing interests

The authors declare that they have no competing interests.

## Authors’ contributions

JCH organized all of the study. HSP, MKK, DKL, KDL, HSL, SWK and ENL participated in the study design and revised the manuscript. SYC drafted and wrote this manuscript. All authors read and approved the final manuscript.
